# Heterogeneity of microsphere distribution in resected liver and tumour tissue following selective intrahepatic radiotherapy

**DOI:** 10.1186/s13550-014-0048-0

**Published:** 2014-09-16

**Authors:** Jonas Högberg, Magnus Rizell, Ragnar Hultborn, Johanna Svensson, Olof Henrikson, Johan Mölne, Peter Gjertsson, Peter Bernhardt

**Affiliations:** Department of Radiation Physics, The Sahlgrenska Academy, University of Gothenburg, SE-41346 Gothenburg, Sweden; Department of Surgery, Sahlgrenska University Hospital, The Sahlgrenska Academy, University of Gothenburg, SE-41346 Gothenburg, Sweden; Department of Oncology, Sahlgrenska University Hospital, The Sahlgrenska Academy, University of Gothenburg, SE-41346 Gothenburg, Sweden; Department of Radiology, Sahlgrenska University Hospital, The Sahlgrenska Academy, University of Gothenburg, SE-41346 Gothenburg, Sweden; Department of Pathology, Sahlgrenska University Hospital, The Sahlgrenska Academy, University of Gothenburg, SE-41346 Gothenburg, Sweden; Department of Clinical Physiology, Sahlgrenska University Hospital, The Sahlgrenska Academy, University of Gothenburg, SE-41346 Gothenburg, Sweden; Department of Medical Physics and Biomedical Engineering, Sahlgrenska University Hospital, The Sahlgrenska Academy, University of Gothenburg, SE-41346 Gothenburg, Sweden

**Keywords:** Radioembolisation, Y-90, SIR, Surgery, Activity heterogeneity

## Abstract

**Background:**

Selective arterial radioembolisation of liver tumours has increased, because of encouraging efficacy reports; however, therapeutic parameters used in external beam therapy are not applicable for understanding and predicting potential toxicity and efficacy, necessitating further studies of the physical and biological characteristics of radioembolisation. The aim was to characterise heterogeneity in the distribution of microspheres on a therapeutically relevant geometric scale considering the range of yttrium-90 (^90^Y) β-particles.

**Methods:**

Two patients with intrahepatic cholangiocarcinoma, marginally resectable, were treated by selective arterial embolisation with ^90^Y resin microspheres (SIRTEX®), followed 9 days post-infusion by resection, including macroscopic tumour tissue and surrounding normal liver parenchyma. Formalin-fixed, sectioned resected tissues were exposed to autoradiographic films, or tissue biopsies of various dimensions were punched out for activity measurements and microscopy.

**Results:**

Autoradiography and activity measurements revealed a higher activity in tumour tissue compared to normal liver parenchyma. Heterogeneity in activity distribution was evident in both normal liver and tumour tissue. Activity measurements were analysed in relation to the sample mass (5 to 422 mg), and heterogeneities were detected by statistical means; the larger the tissue biopsies, the smaller was the coefficient of variation. The skewness of the activity distributions increased with decreasing biopsy mass.

**Conclusions:**

The tissue activity distributions in normal tissue were heterogeneous on a relevant geometric scale considering the range of the ionising electrons. Given the similar and repetitive structure of the liver parenchyma, this finding could partly explain the tolerance of a relatively high mean absorbed dose to the liver parenchyma from β-particles.

## Background

Primary and metastatic liver tumours present a significant clinical problem, when radical surgery, the only curative option, is not possible because of extensive growth in connection with vital structures, even without extra-hepatic growth. Chemotherapy has limited efficacy [1-3], and external beam radiotherapy, including stereotactic techniques, is hampered by a normal liver tissue tolerance that is less than needed for tumour eradication [4,5]. Other techniques used for local destruction of liver tumours include radiofrequency or ultrasound ablation and are moderately successful [6-8]; thus, new treatment modalities are urgently needed. Because liver malignancies are supplied mainly from the hepatic artery, in contrast to portal vein supply to the liver parenchyma [9], selective intra-arterial interventions have been used, including arterial chemoembolisation [10] and radioembolisation.

The evidence favouring one technique over the other is weak. However, it seems clear that with present radioembolisation techniques using yttrium-90-labelled (^90^Y-labelled) microspheres selectively infused via the hepatic artery, tumour reduction can be achieved with relatively little radiation-induced hepatic damage in spite of mean liver doses far exceeding those tolerated in external beam therapy [9,11,12]. Knowledge of the physical and biological background is meagre; not least the explanation of the relatively high tolerance for ionising radiation of the normal liver tissue, but it is probably related to the low dose rate of radionuclide therapy and dose heterogeneity, allowing for regeneration from low-dose regions in the normal liver parenchyma.

The possible sparing effect of the low dose rate has been theoretically investigated by Cremonesi et al. [13]. They showed a minor sparing effect in regard to biologically effective doses (BEDs). In contrast, large non-uniform microsphere distributions have been observed in surgically removed tissue samples after radioembolisation [14-17]. However, no clear quantitative description of the non-uniformity has been presented from this limited number of studies and the main focus has been tumour tissue rather than normal liver parenchyma. Despite the unknown small-scale distribution of microspheres, Gulec et al. presented a detailed Monte Carlo modelling approach for performing small-scale dosimetry [18]. They used millions of closed-packed hexagonal lobules (radius 0.6 mm and length 1.5 mm) to model the liver parenchyma, and the microspheres were uniformly distributed in the portal tracts in the corners of the lobules. Despite the long range of ^90^Y electrons (max range, 11 mm; mean range, 2.5 mm) [19], a more than twofold difference in the absorbed doses to the fine structures was obtained; the cross-irradiation would only partly smooth the dose distribution. Therefore, it can be anticipated that a variable distribution of microspheres in the portal tracts will create a primarily non-uniform dose distribution in the liver parenchyma.

To investigate the question of heterogeneity, here we evaluated activity distributions on biopsies taken from resected tissue samples pre-surgically treated with resin microspheres (SIR-Spheres®).

## Methods

### Patients

Two patients were selected with cholangiocarcinoma that was confined to the liver but only marginally resectable because of proximity to critical vascular and biliary structures. They underwent radioembolisation followed by marginal resection, with the rationale to reduce the risk of local recurrence at the resection margins.

The study was carried out in compliance with the Declaration of Helsinki and has been approved by the Regional Ethical Review Board in Gothenburg, Sweden. Both patients have given written consent to the treatment.

Patient 1 (Pt 1), a female age 33 years, had a tumour mass of approximately 770 and 180 g, in the left and right liver lobes, respectively, with normal liver tissue of approximately 100 and 1,200 g in the respective lobes, as determined from consecutive CT sections. Patient 2 (Pt 2), a female age 62 years, had a tumour mass of 37 and 56 g in the left and right lobes, respectively, close to the vena cava, liver veins, and the portal tract, with corresponding normal liver parenchyma of 700 and 1,350 g, respectively. Surgery was performed on both patients on the ninth day after radioembolisation.

### Radioembolisation

The patients underwent selective hepatic artery angiography followed by infusion of technetium-99m-labelled macro-aggregated albumin (^99m^Tc-MAA) to obtain information about distribution to enable subsequent activity prescription as well as ruling out excessive pulmonary shunting [20]. When calculating BED, the following parameters were chosen: *α*/*β* = 2.5 Gy for the liver parenchyma and *α*/*β* = 10 Gy for the tumours. This is in accordance with the calculations by Cremonesi et al. [13] as well as with the recommendations given by Dale and Jones [21] for tumour types with limited documentation on radiosensitivity as well as repopulation rate, e.g. cholangiocarcinoma [22-25].

Two weeks later, the hepatic artery was recannulated for infusion of the ^90^Y-labelled SIR-Spheres (Sirtex Medical Limited, North Sydney, Australia). The microspheres consist of polymer resin with a mean diameter of 30 μm (20 to 60 μm), aggregated with the beta emitter ^90^Y, with an approximate activity of 50 Bq (40 to 70 Bq) per sphere. The mean and maximum ranges of the electrons (mean energy, 0.934 MeV) in soft tissues are 2.5 and 11 mm, respectively. The half-life of ^90^Y is 64 h, so that only 9% to 10% of the injected activity would remain at the day of surgery. The number of spheres injected during approximately an hour, together with 30 to 40 ml distilled water, was about 50 billion (2.4 GBq) for Pt 1 and 30 billion (1.6 GBq) for Pt 2. The construction of the application device will minimise the risk of microsphere aggregations within the infusate [26-28].

### Surgery

The patients were re-admitted for surgery on the ninth day post-radioembolisation. The surgical procedures, performed by an experienced specialist (MR), targeted removal of the macroscopic tumour mass to include a rim of surrounding normal liver tissue when possible. An ultrasonic cavitron aspirator was used for dissection. Radiation safety issues during surgery were studied and have been previously reported [29].

### Sample preparation and autoradiography

The resected tissue was immediately immersed in 10% neutral formaldehyde solution for 48 h and then machine sliced in sections approximately 1 to 8 mm thick for Pt 1 and 1 to 2 mm thick for Pt 2. Every other slice was covered in double layers of 0.2-mm plastic film and exposed to autoradiography films (Amersham Hyperfilm® MP, GE Healthcare, Uppsala, Sweden) in cassettes (for 5 h, 5 days after surgery for Pt 1 and 2 h, 3 days after surgery for Pt 2). The remaining slices were punched in various dimensions (diameters 3, 4, 6 and 8 mm): 8 to 422 mg for Pt 1 and 5 to 102 mg for Pt 2. The biopsies were weighed and immersed in 1 ml formaldehyde solution (formalin) in plastic vials with an inner diameter of 9 mm and a wall thickness of 1 mm. The activity was measured from the *bremsstrahlung* in an automatic gamma well counter (Wizard® 1480, PerkinElmer, Waltham, MA, USA), with activity calibrated for ^90^Y in the same vial type and liquid volume.

### Analysis of heterogeneity

Apart from autoradiography, the coefficient of variation (CV) of the activity concentration distribution in biopsies was studied. CV decreasing with increasing biopsy volumes sizes shows a pattern of repetitive heterogeneity, consistent with the repetitive structure of the liver parenchyma. CVs lacking this trend (sometimes decreasing, sometimes increasing with increasing biopsy volume) show a chaotic, irregular unstructured heterogeneity that cannot be extended to a larger volume. A large variance of biopsy volumes within the sample mass group could also cause this effect. A strong negative trend with increasing biopsy volume would indicate a heterogenic pattern with a larger basic element size than the compared volumes. A weak negative trend of consistently small CV values indicates that the heterogenic pattern probably has smaller elements than the biopsy volumes investigated. The explanation for this is that almost all the expected variation due to heterogeneity will be found within each biopsy and the variance within a sample of biopsies would be explained by systematic variations (gradients in activity concentrations) rather than variations caused by inter-regional structure differences causing different distribution patterns. The skewness (SK) of the distribution was calculated with the adjusted Fisher-Pearson standardised moment coefficient [30]:

1 $$ \mathrm{S}\mathrm{K}=\frac{n}{\left(n-1\right)\left(n-2\right)}{{\displaystyle \sum_{i=1}^n\left(\frac{x_i-\overline{x}}{s}\right)}}^3 $$

A negative skew indicates many biopsies of low activity (cold spots more common than hot spots), and a positive skew indicates the opposite, many biopsies of high activity (hot spots more common than cold spots). To study normal liver tissue by light microscopy, 10 serial sections, each 20 μm thick, were prepared from each of 6 (circular diameters of 4 to 8 mm) normal liver biopsies of Pt 2, resulting in a total of 60 slices. The biopsies were paraffin embedded, sectioned and stained with haematoxylin and eosin. Microspheres were counted and grouped by studying sequential sections under light microscopy.

## Results

SPECT/CT studies from the ^99m^Tc-MAA infusions disclosed a pulmonary shunting of 3.0% and 3.5% for Pt 1 and Pt 2, respectively. The tumour-to-normal liver activity concentrations (TNCs), averaging voxel levels in the entire tumours as well as entire normal liver tissues, were calculated at 2.7 and 3.8 in Pt 1 and Pt 2, respectively. The CVs were 0.29 (normal) and 0.42 (tumour) for Pt 1 and 0.77 (normal) and 0.089 (tumour) for Pt 2.

From the pre-therapy studies, the prescribed activities to be delivered would result in expected average doses and BEDs to normal liver of 22 (BED = 29) Gy and 33 (BED = 49) Gy for Pt 1 and Pt 2, respectively. For practical reasons, the doses were averaged over both the portions of normal tissue planned to be resected (tumour margins) as well as the living liver tissue remaining in the patient after surgery. The expected (if unresected) doses to tumours (in accordance with TNC values reported above) were calculated to 59 (BED = 67) Gy and 125 (BED = 161) Gy for Pt 1 and Pt 2, respectively.

Based on the measured activity in biopsies, the average absorbed dose to normal liver parenchyma was calculated to 31 (BED = 45) Gy (620 Bq/mg) for Pt 1 and 52 (BED = 93) Gy (1,040 Bq/mg) for Pt 2. The corresponding calculation based on tumour biopsies resulted in 100 (BED = 123) Gy (2,000 Bq/mg) for Pt 1 and 150 (BED = 202) Gy (3,050 Bq/mg) for Pt 2. Thus, based on the limited sample volumes, the TNCs were 3.2 and 2.9 for Pt 1 and Pt 2, respectively.

**Figure 1:**
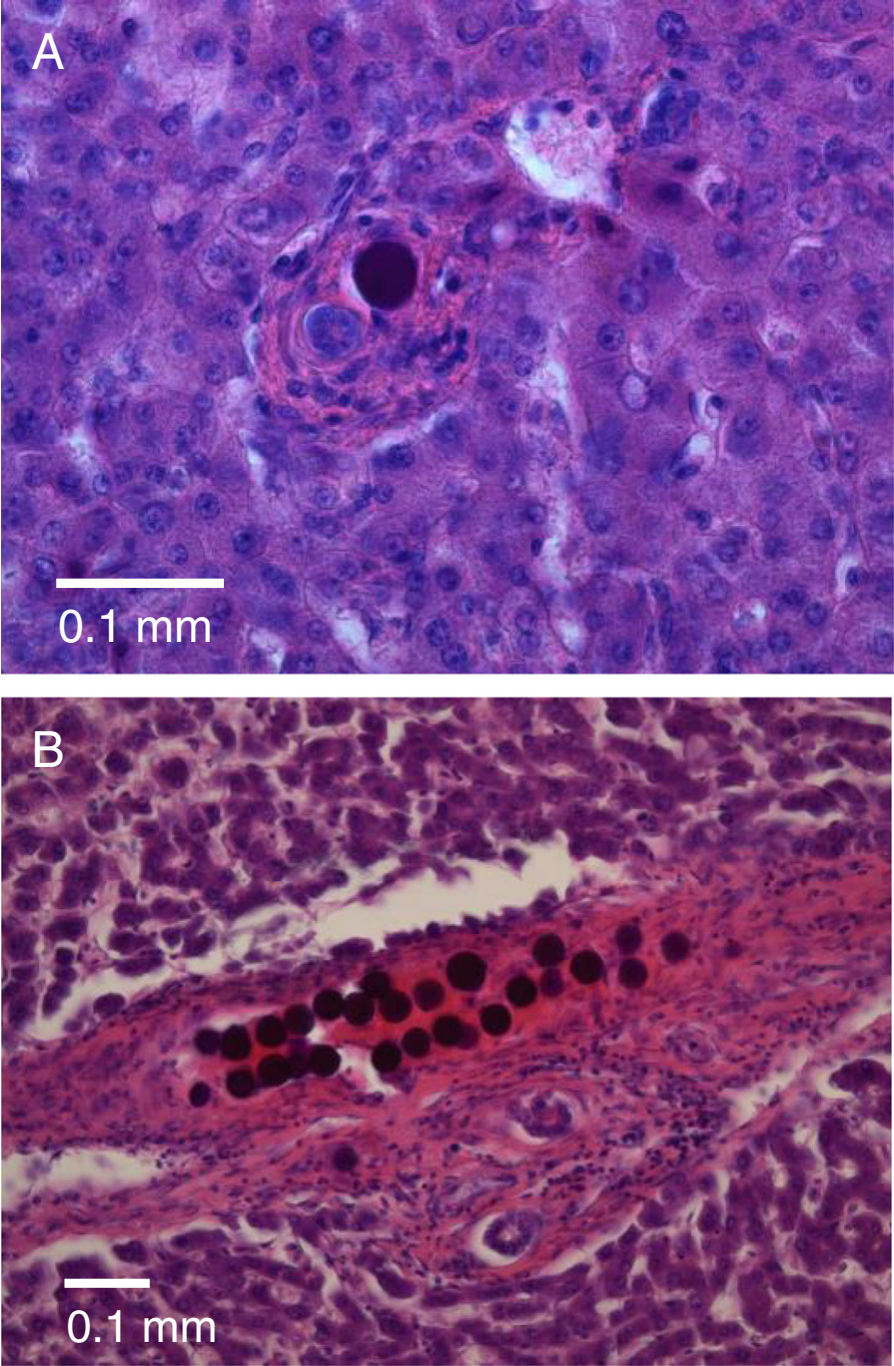
**Light microscopy images illustrating the three main types of microsphere aggregations found in Pt 2. (A)** Single isolated sphere in an arteriole in a small portal tract, magnified × 200. **(B)** A large cluster of 27 spheres in a relatively large portal tract with a wide arteriole, ×100. Based on adjacent slices, this cluster was part of a cluster of 36 spheres.

**Figure 2:**
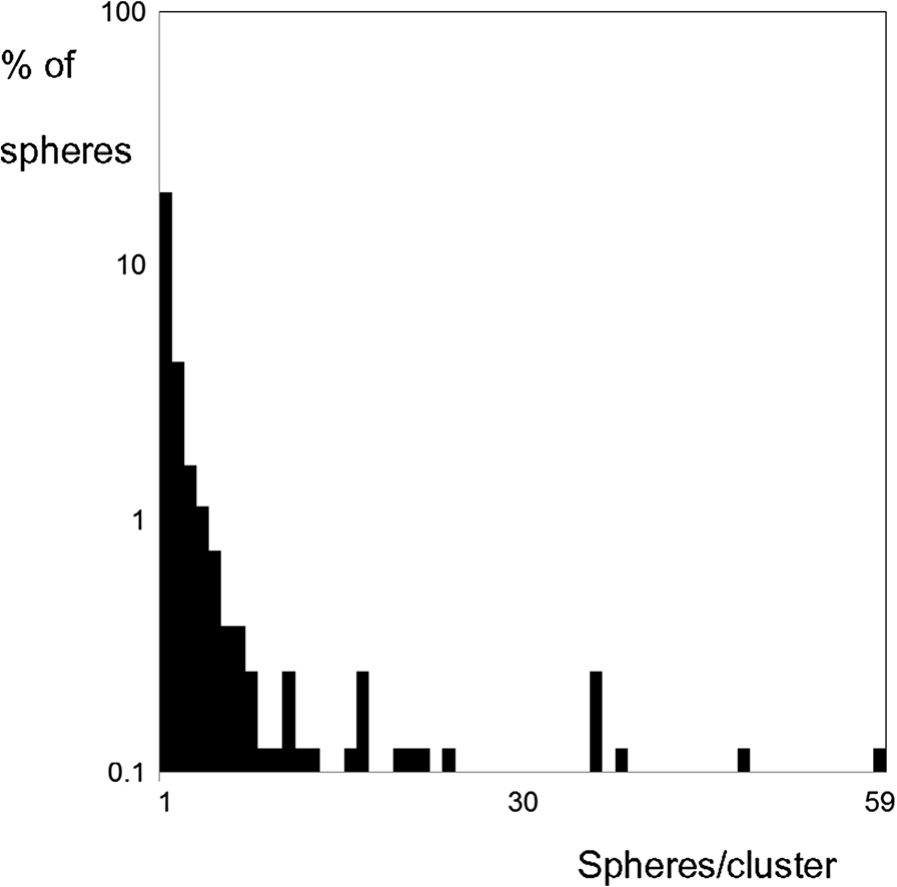
**Histogram reflecting the distribution of spheres observed by microscopy in normal liver parenchyma of Pt 2.** Six biopsies from Pt 2, with a total volume of 36 mm^3^, divided into 60 slices were investigated. Bars show the number of clusters found with each size, i.e. spheres per cluster. Fifty-three percent of all 793 spheres found were located within any of the larger clusters (8 to 59 spheres/cluster).

In Pt 2, different clustering patterns of the microspheres were found with light microscopy, with several clusters distributed through more than one serial section. The seven largest clusters, of the type shown in Figure 1B, ranged 22 to 59 microspheres when aligning serial adjacent slices. Of the 240 locations found with single or several microspheres, 154 were isolated single spheres (Figure 1A), i.e. at a distance >200 μm (7 sphere diameters) from the nearest neighbouring sphere. This distance was chosen as it was consistent with the largest portal tract found and therefore two single spheres would not occupy the same portal tract. Thus, 64% of all locations contained single isolated spheres, representing only 19% of the total number of spheres; 53% of all 793 spheres were located within any of the 19 largest clusters (7% of the locations), ranging 8 to 59 spheres per cluster (Figure 1B). The mean size of each location was 3.3 spheres, with a CV of 2.1 and SK of 4.9. The distribution of spheres is shown in Figure 2. The total activity found in the six biopsies, divided by their total mass, showed an activity concentration of 1,550 Bq/mg. The 793 spheres (50 Bq per sphere) found in the 60 sections with a total volume of 36 mm^3^ showed an activity concentration of 1,120 Bq/mg (28% lower).

### Autoradiography

**Figure 3:**
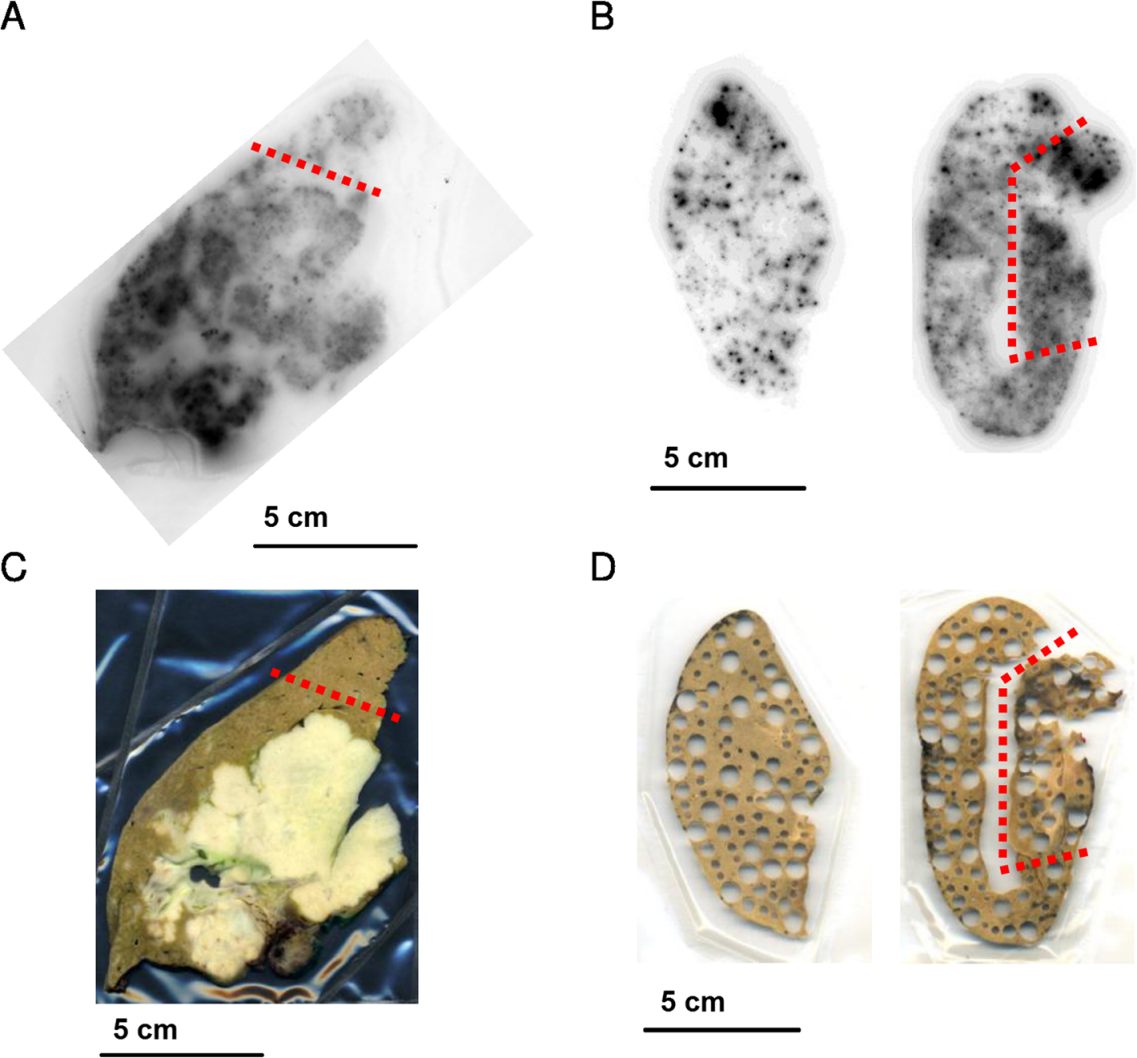
**Schematic overview of tissue samples and radiological methods. (A, C)** Pt 1. **(B, D)** Pt 2. **(A)** and **(C)** show the same tissue slice, with radiography in **(A)** and a photo scan in **(C)**. The small region of **(A)** and **(C)**, limited by the dotted red line, is normal liver parenchyma with no visual signs of tumour involvement, as opposed to the larger tissue region on the other side of the line. The images to the left in **(B)** and **(D)** show autoradiography and biopsy punching of normal liver tissue. The images to the right in **(B)** and **(D)** show likewise for tumour and normal liver parenchyma without tumour involvement but adjacent to the tumour. The dotted red line in **(B)** and **(D)** limits the smaller tumour tissue region from the adjacent normal liver parenchyma without visual signs of tumour involvement. **(D)** shows how several small tissue biopsies of different sizes have been punched out, from the tissue slices being examined with autoradiography in **(B)**, to measure their activity concentration. For Pt 1, samples were also punched out from the slice shown in **(C)** but after photography.

Images acquired through autoradiography, performed on resected tumour and normal liver parenchyma, are shown in Figure 3. The images indicate a heterogenic pattern, with several hot and cold spots of radioactivity and a generally higher activity concentration in tumour tissue.

### Detector measurements

Tables 1, 2 and 3 show statistical data for the radioactivity distribution of Pt 1. No clear decreasing or increasing trends are observable for CVs or SKs; however, the CVs were consistently lower for the left lobe, showing a higher mean activity concentration.

**Table 1:** **Activity distributions in normal liver tissue from the left lobe of Pt 1**

	**Small mass**	**Medium mass**	**Large mass**	**All**
Mass (mg)	8 ≤ *m* ≤ 41	47 ≤ *m* ≤ 76	80 ≤ *m* ≤ 422	8 ≤ *m* ≤ 422
$\overline{m}$ (mg)	20	63	210	100
*n*	11	11	12	34
$\overline{\mathrm{Spheres}}/\mathrm{biopsy}$	1,200	3,300	9,200	4,400
$\overline{A}/\overline{m}$ (Bq/mg)	3,100	2,600	2,200	2,200
CV	0.45	0.46	0.41	0.46
SK	−0.35	−0.52	−0.12	0.058

CV, coefficient of variation; $\overline{m}$, mean mass of *n* tissue biopsies; $\overline{\mathrm{Spheres}}/\mathrm{biopsy}$, average number of spheres per biopsy; $\overline{A}/\overline{m}$, mean activity concentration; SK, skew of the distribution.

**Table 2:** **Activity distributions in normal liver tissue from the right lobe of Pt 1**

	**Small mass**	**Medium mass**	**Large mass**	**All**
Mass (mg)	12 ≤ *m* ≤ 62	63 ≤ *m* ≤ 125	128 ≤ *m* ≤ 391	12 ≤ *m* ≤ 391
$\overline{m}$ (mg)	39	82	240	120
*n*	22	20	20	62
$\overline{\mathrm{Spheres}}/\mathrm{biopsy}$	510	740	2,200	1,200
$\overline{A}/\overline{m}$ (Bq/mg)	670	450	470	480
CV	0.63	0.67	0.57	0.65
SK	0.27	2.8	1.7	1.2

CV, coefficient of variation; $\overline{m}$, mean mass of *n* tissue biopsies; $\overline{\mathrm{Spheres}}/\mathrm{biopsy}$, average number of spheres per biopsy; $\overline{A}/\overline{m}$, mean activity concentration; SK, skew of the distribution.

**Table 3:** **Activity distributions in tumour tissue of Pt 1**

	**Liver lobe**			
	**Left**		**Right**	
Mass (mg)	15 ≤ *m* ≤ 26	54 ≤ *m* ≤ 104	49 ≤ *m* ≤ 73	76 ≤ *m* ≤ 90
$\overline{m}$ (mg)	21	75	60	82
*n*	16	16	9	9
$\overline{\mathrm{Spheres}}/\mathrm{biopsy}$	1,500	3,000	1,100	980
$\overline{A}/\overline{m}$ (Bq/mg)	3,600	2,000	910	610
CV	0.49	0.75	0.66	0.51
SK	−0.46	0.82	1.0	0.64

CV, coefficient of variation; $\overline{m}$, mean mass of *n* tissue biopsies; $\overline{\mathrm{Spheres}}/\mathrm{biopsy}$, average number of spheres per biopsy; $\overline{A}/\overline{m}$, mean activity concentration; SK, skew of the distribution.

**Figure 4:**
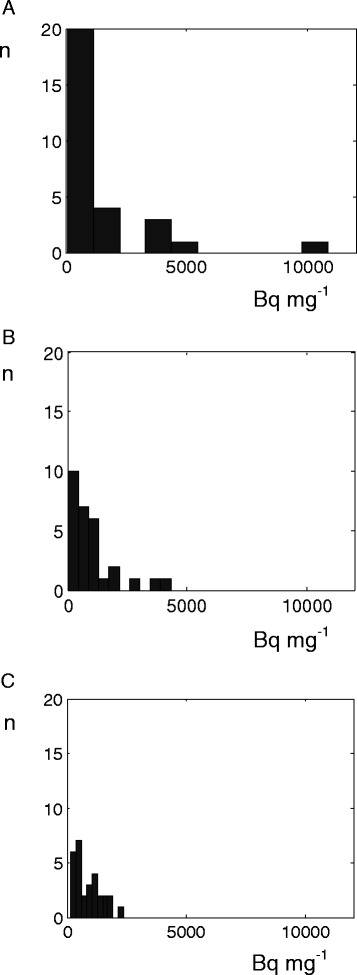
**Activity concentration histograms reflecting distributions in biopsies of normal liver parenchyma of Pt 2.** Bars show the number of samples for each interval. Each histogram is split into ten activity intervals, giving different bar widths depending on the maximum activity identified. The histograms reflect biopsies at a relatively large distance from the tumour but with different mass intervals: **(A)** masses 6 to 14 mg, **(B)** masses 15 to 39 mg and **(C)** masses 40 to 91 mg.

Tables 4, 5 and 6 show the radioactivity distribution data for Pt 2. Tables 4 and 5 show CV and SK decreasing with increasing mass. A comparison of CV levels between the tables shows a higher CV for normal liver parenchyma at some distance from the tumour, compared to adjacent to the tumour, consistent with differing mean activity concentrations. Figure 4 shows histograms of the data presented in Table 4.

**Table 4:** **Activity distributions in normal liver tissue far away from the tumour of Pt 2**

	**Small mass**	**Medium mass**	**Large mass**	**All**
Mass (mg)	6 ≤ *m* ≤ 14	15 ≤ *m* ≤ 39	40 ≤ *m* ≤ 91	6 ≤ *m* ≤ 91
$\overline{m}$ (mg)	10	27	66	35
*n*	29	26	29	84
$\overline{\mathrm{Spheres}}/\mathrm{biopsy}$	300	590	1,200	740
$\overline{A}/\overline{m}$ (Bq/mg)	1,500	1,100	900	1,000
CV	1.4	1.0	0.63	1.3
SK	3.0	1.8	0.82	4.0

CV, coefficient of variation; $\overline{m}$, mean mass of *n* tissue biopsies; $\overline{\mathrm{Spheres}}/\mathrm{biopsy}$, average number of spheres per biopsy; $\overline{A}/\overline{m}$, mean activity concentration; SK, skew of the distribution.

**Table 5:** **Activity distributions in normal liver tissue adjacent to the tumour of Pt 2**

	**Small mass**	**Medium mass**	**Large mass**	**All**
Mass (mg)	5 ≤ *m* ≤ 14	16 ≤ *m* ≤ 41	43 ≤ *m* ≤ 102	5 ≤ *m* ≤ 102
$\overline{m}$ (mg)	10	27	69	35
*n*	26	24	25	75
$\overline{\mathrm{Spheres}}/\mathrm{biopsy}$	380	920	2,300	1,200
$\overline{A}/\overline{m}$ (Bq/mg)	1,900	1,700	1,700	1,700
CV	0.81	0.58	0.45	0.64
SK	1.8	1.2	0.69	1.9

CV, coefficient of variation; $\overline{m}$, mean mass of *n* tissue biopsies; $\overline{\mathrm{Spheres}}/\mathrm{biopsy}$, average number of spheres per biopsy; $\overline{A}/\overline{m}$, mean activity concentration; SK, skew of the distribution.

**Table 6:** **Activity distributions in tumour tissue of Pt 2**

	**Small mass**	**Medium mass**	**Large mass**	**All**
Mass (mg)	5 ≤ *m* ≤ 11	12 ≤ *m* ≤ 37	38 ≤ *m* ≤ 95	5 ≤ *m* ≤ 95
$\overline{m}$ (mg)	8.4	23	65	32
*n*	14	14	13	41
$\overline{\mathrm{Spheres}}/\mathrm{biopsy}$	690	2,100	3,100	1,900
$\overline{A}/\overline{m}$ (Bq/mg)	4,100	4,500	2,400	3,000
CV	0.50	0.71	0.36	0.65
SK	0.78	2.9	−1.3	2.8

CV, coefficient of variation; $\overline{m}$, mean mass of *n* tissue biopsies; $\overline{\mathrm{Spheres}}/\mathrm{biopsy}$, average number of spheres per biopsy; $\overline{A}/\overline{m}$, mean activity concentration; SK, skew of the distribution.

Tumour tissue analysis revealed no clear trend of decrease or increase with increasing biopsy volumes for CVs and SKs (Table 6).

## Discussion

In this study, surgical resection of tumours enabled a detailed investigation of microsphere distribution. To avoid haemorrhage and dissection difficulties along critical structures, surgery was performed before radiation-induced inflammatory reactions developed. Late surgery after inflammatory decline might have increased the risk for extensive fibroses.

The current autoradiogram results, showing irregular distributions and varying sizes of hot and cold spots of radioactivity, are in line with previous studies, mainly involving light microscopy evaluation of tissue samples collected months after injection [14-17]. However, the previous and current studies are of limited value for describing a full volumetric distribution of microspheres, because serially aligned sections have to be prepared and carefully analysed, which was done in limited volume in this study. We also applied a less time-consuming new approach for analysing clustering size and its distributions.

Our approach to studying the geometric pattern of non-uniformity relied on measurements of activity concentration in tissue samples of various sizes. With this approach, the SK and CV should decrease with increasing biopsy size for heterogenic patterns with larger basic elements than the biopsy sizes.

The difference between TNCs, comparing imaging and biopsy measurements, can be explained by the fact that the latter method excludes averaging over the tissue left in the patient after surgery and that tumour tissue dominated the resected volume of Pt, whereas resected normal liver parenchyma dominated for Pt 2.

In Pt 1, the mean activity concentration decreased with increasing masses, but CVs and SKs did not follow the expected trend. These results indicate that the mass groups probably were too wide and mean masses may have been too large. Mass homogeneity of each mass group thus appears statistically important; furthermore, the number of biopsies might have been too low.

The normal liver parenchyma consists of a very well structured distribution of more than one million lobules and associated portal tracts. Each lobule is associated with six portal tracts, but because of the hexagonal arrangement of lobules, three lobules share each portal tract. Therefore, there are no more than two portal tracts per lobule throughout the parenchyma. A lobule weighs around 2 mg, so there is about one portal tract per milligram of liver tissue [31]. Considering the portal tracts as the target site for microspheres, the number of target sites in the sample sizes from Pt 1 could range from 8 to 62 in the smallest sample groups to over 400 in the largest. To identify any spatial variation in cluster frequency, the sample sizes must include few targets with a low variation in mass.

The results from Pt 2 with many biopsies and smaller masses with a low variation in mass showed a clear trend consistent with the heterogeneity of normal liver parenchyma, with strictly decreasing CV and SK with increasing masses. The strong decreasing trend of the CV indicates that the basic element of the heterogenic pattern is larger than or similar in size to the biopsies in the group with the largest masses (about 70 mg). The decreasing positive SK indicates a rather frequent and repetitive presence of hot spots of radioactivity, i.e. microsphere clusters or regional gatherings. The small sample investigated by light microscopy showed a similar and slightly higher CV (2.1) and SK (4.9) than was identified by activity measurements on the sample group of origin (CV of 1.3 and SK of 4.0; Table 4). These findings seem to agree with the repetitive microscopic vascularisation structure of the liver. As only 8% of the total volume of the six biopsies selected for microscopic studies were in fact investigated in microscope, it is not surprising that the activity concentration differed with 28% comparing sphere counting in microscope with detector measurements on the same six biopsies.

Comparison of the two normal tissue regions showed a lower CV with higher activity concentration and similar masses. This finding indicated decreasing heterogeneity with increased activity concentration.

In contrast to normal liver parenchyma, tumour tissue architecture is unstructured, with scattered fibrotic and necrotic areas that obviously influence the statistical results. The current inconclusive results for tumour tissue are included only to highlight how our method, when applied correctly (e.g. for Pt 2), could distinguish a structured heterogenic distribution (normal liver parenchyma) from a more chaotic heterogenic distribution (tumour). These findings are in accordance with those of Tveit et al. who studied the regional perfusion *ex vivo* in human kidney specimens, including carcinomas. They found a homogeneous high perfusion in the well-structured renal cortex in contrast to the highly inhomogeneous perfusion in the tumour tissue [32]. The results for normal liver tissue are based on a comparatively large number of tissue biopsies: 96 from Pt 1 and 159 biopsies of Pt 2, supporting our conclusions.

Limited data on microsphere distribution in patients are available. Some efforts have targeted dosimetric calculations, and studies mainly have been light microscopy investigations of tissue samples collected months after administration [15-17,33,34]. The reported results are consistent with ours, showing large heterogeneities in microsphere distribution arising from clustering effects and also heterogeneity patterns that are more prominent in normal liver tissue than in tumour tissue.

Gulec et al. simulated dose distributions in the context of liver tissue microanatomy and showed different mean absorbed doses to different tissue structures [18]. However, they noted that they never intended to consider the expected heterogeneities in the distribution of microspheres; thus, the assumed homogeneous distribution resulted in different mean doses between different structure types, but basically no variance *within* structure types. Applying our findings of heterogeneities on a large scale could be clinically valuable, comparing them to the size of individual liver lobules used as the fundamental unit in their simulations and showing a more realistic dose variance within specific structure types. Our findings suggest that some lobules may receive very few or no microspheres whereas others may receive large clusters or strings of spheres. Indeed, larger clusters of microspheres might be stuck within larger arterioles, limiting the mean absorbed dose to finer liver structures, such as within the liver lobules.

Recently, Walrand et al. simulated different arterial tree models, varying the parameters affecting the distribution of glass microspheres [35]. Their results showed heterogeneities and clustering of spheres due to stochastic and systematic variations of the blood flow. Their clusters tended to be much smaller. The majority of the spheres were found within clusters of two to three spheres, about 30% to 40% were found to be single spheres, 10% of the spheres were found within clusters of four to five spheres and only a few percent within cluster sizes of seven to ten spheres. The explanation for this could be that the 25 times lower specific activity per resin sphere, as compared to glass spheres, and thus a much higher number of resin spheres injected, tends to create larger clusters, for statistical reasons. This would be consistent with the results from our study, with the majority of the spheres trapped within cluster sizes of 8 to 59 spheres. It is also possible that the more extensive embolisation caused by higher numbers of resin spheres has a more dramatic effect on the arterial circulation, causing larger differences in blood flow and thus larger inter-regional variations in distributions of microspheres, making the mentioned model less applicable for resin spheres.

We studied heterogeneity on a scale slightly larger than the mean range of β^−^ electrons from ^90^Y. Interestingly, the CVs for activity distributions are higher within limited regions, as measured on biopsies, than in entire tissue volumes, as measured with ^99m^Tc-MAA. Even though the ^99m^Tc-MAA gamma radiation is smeared out, the homogenising effect of crossfire between microspheres will not smooth heterogeneity at this scale (the aim in treating tumour tissue, but unwanted in normal liver parenchyma), as the mean range of the ^90^Y beta radiation is only 2.5 mm. The statistical methods used here demonstrate activity heterogeneity at a therapeutically relevant geometric scale, which might be useful for protocol optimisation. In their modelling study, Cremonesi et al. showed that liver tissue might be spared by fractionated administration of microspheres [13]. In such calculations, however, the sparing effect arises only from a decreased dose rate. Our results indicate that the heterogeneity of microsphere trapping between fractions might heighten the sparing effect of fractionation protocols.

To obtain a more precise dosimetric distribution from our results, the heterogenic distribution of activity must be applied to a detailed 3-D activity distribution matrix model, convolved with a 3-D beta dose kernel matrix, to plot reliable dose-distribution histograms for input in response models such as the BED model.

## Conclusions

Tissue activity distributions in normal tissue were heterogeneous on a relevant geometric scale considering the mean range of the ionising electrons. Given the similar and repetitive structure of the liver parenchyma within and between patients, the detected heterogeneities in normal liver parenchyma can partly explain the survival of normal tissue in regions receiving a substantially higher mean absorbed dose than could be tolerated with more homogeneous dose distributions resulting from external radiation techniques.
